# Successful Treatment with Propranolol in a Patient with a Segmental Hemangioma: A Case Report

**DOI:** 10.5505/tjh.2012.37233

**Published:** 2012-06-15

**Authors:** Serhan Küpeli, Derya Çimen, Begül Yağcı Küpeli

**Affiliations:** 1 Diyarbakır Children’s Diseases Hospital, Department of Pediatric Oncology, Diyarbakır, Turkey; 2 Diyarbakır Children’s Diseases Hospital, Department of Pediatric Cardiology, Diyarbakır, Turkey; 3 Dicle University, School of Medicine, Department of Pediatric Oncology, Diyarbakır, Turkey

**Keywords:** Hemangioma, Segmental hemangioma, Subglottic hemangioma, Propranolol

## Abstract

The treatment of hemangiomas in infancy may be associated with significant morbidity. In addition to morbidity, an objective response cannot be obtained because of the absence of targeted therapeutic options. Herein, we present an infant with a segmental hemangioma and marked glucocorticoid toxicity due to prior steroid therapy that was successfully treated with propranolol. Propranolol was tolerated well and no side effects were observed during the treatment. The only problem to occur was disease recurrence following the withdrawal of propranolol at age 13 months, which was not within the age of spontaneous regression (generally considered as >18 months).

## Abstract (tr)

Süt çocukluğu döneminde hemanjiyomların tedavisinde önemli yan etkilerle karşılaşılabilmektedir. Hedefe yönelik tedavi seçeneklerinin olmayışı nedeniyle de karşılaşılan morbiditenin yanısıra objektif cevap elde edilmesinde de güçlük çekilmektedir. Bu yazıda segmental hemanjiyomu olan ve steroid tedavisine ikincil ağır glukokortikoid toksisitesi gelişmiş bir süt çocuğunda propranolol tedavisi ile elde edilen başarılı sonuçlar sunulmuştur. Tedavi süresince propranolol iyi tolere edilmiş ve herhangi bir yan etki görülmemiştir. Hasta 13 aylık iken propranolol tedavisinin kesilmesiyle hemanjiyomda tekrarlama görülmüş ve propranolol tekrar başlanmak zorunda kalınmıştır. Hemanjiyomlarda spontan regresyon yaşının 18 aylıktan sonra olduğu düşünülmektedir.

## INTRODUCTION

Hemangioma is the most common tumor in infants. Although the first-line treatment for superficial and lifethreatening hemangiomas is systemic corticosteroids, side effects limit their long-term use. Recent case reports on the successful treatment of hemangiomas with the β-blocking agent propranolol have been published [1,2,3,4,5,6,7,8]. Herein, we describe an infant with a segmental hemangioma that was successfully treated with propranolol, which was initially administered to treat tachycardia.

## CASE REPORTS

A 5-month-old female presented with purulent discharge on the neck, red swelling on the face, and occasional attacks of dyspnea that began at age 1 month. The patient’s medical history included multiple hemangiomas on her face at birth and stridor due to a subglottic hemangioma observed via laryngoscopic examination at age 2 months. At age 3 months, she was started on methylprednisolone (MPZ) 30 mg·kg[u]–1[/u]·d[u]–1[/u] by doctors at another hospital due to airway obstruction. The treatment plan at another hospital was to taper MPZ, but as the dyspnea and swelling on the face and neck recurred at the end of 2 weeks, the steroid treatment could not terminated. We have an approval from institutional ethical committee. We have a written informed consent signed by parents of the patient at the beginning of the propranonol treatment. 

**Figure 1:**
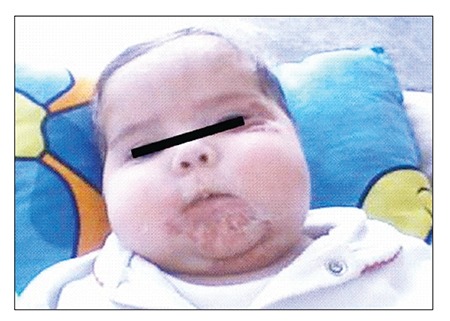
Hemangiomas and cushingoid appearance (pre-treatment).

Physical examination showed hemangiomas on the neck, bilateral preauricular area, mandible, anterior region of the tongue, and left periorbital region (Figure 1). Cushingoid apperence and purulent discharge were observed on the ulcerated hemangioma in the cervical region. Heart rate was 180 bpm. Electrocardiogram (ECG) showed sinus tachycardia, and 1° mitral insufficiency was observed with echocardiography (ECHO). Abdominal ultrasonography, chest X-ray, and cranial MRI—performed to exclude associated multiple anomalies including PHACES syndrome— were normal. Oral propranolol 2 mg·kg[u]–1[/u]·d[u]–1[/u] was started for the treatment of the hemangiomas, considering that the patient’s sinus tachycardia would benefit as well. Topical antibacterial was used for the infection on the patient’s neck and MPZ was reduced to 5 mg·kg[u]–1[/u]·d[u]–1[/u]. 

**Figure 2:**
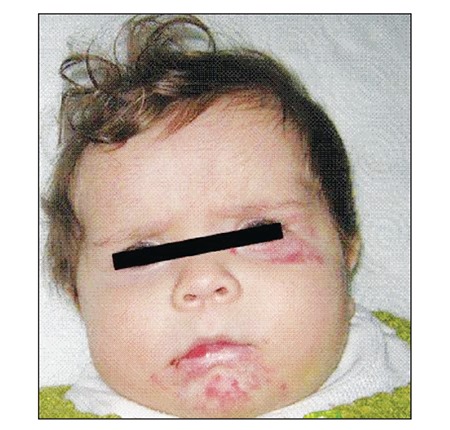
Hemangiomas on the face following propranolol treatment.

Fifteen days after the initiation of oral propranolol treatment the family reported that the infant had no difficulty breathing and on examination we determined no purulent discharge and partial regression in hemangiomas (Figure 2). MPZ was further reduced to 3 mg·kg[u]–1[/u]·d[u]–1[/u], and then tapered to half of the previous dose 1 week apart and stopped over 1 month. The propranolol dose was adjusted monthly according to the patient’s body weight. Propranolol was withdrawn at age 13 months, as the hemangiomas on the patient’s face, neck, and tongue completely regressed, and there was no evidence of respiratory distress. The patient was brought again to hospital 15 d after the withdrawal of propranolol with dyspnea and stridor and hyperemia on previous lesions. Propranolol was started at the dose of 2 mg·kg[u]–1[/u]·d[u]–1[/u] and given until age 20 months. At the time this report was written the patient was aged 22 months and symptom-free.

## DISCUSSION

Hemangiomas in infants present with a diverse spectrum of clinical features—from non-symptomatic superficial lesions to life-threatening giant masses. Symptomatic airway and visceral hemangiomas can be observed in infants with extensive facial hemangiomas. They are the most common vascular tumor of childhood, with an incidence rate of 1.1%-2.6% among full-term neonates [2]. The lesions are often invisible or very small at birth, and exhibit rapid growth during the first 3-6 months of life, followed by regression especially after the first year. Extensive cervicofacial hemangiomas can be part of PHACES syndrome, which is characterized by posterior fossa malformations, hemangiomas, arterial anomalies, coarctation of the aorta and other cardiac defects, eye abnormalities, and sternal defects. 

Systemic corticosteroids have been the mainstay of treatment for hemangiomas since 1960’s. Oral or intravenous MPZ, and prednisone are effective for shrinking hemangiomatous lesions in infants; however, the use of systemic corticosteroids is limited due to the potential for numerous side effects, including growth delay, cushingoid appearance, behavioral changes, irritability, gastrointestinal disturbance, hypertension, adrenal suppression, and compromised immunity [1]. Cushingoid appearance and delayed healing of the cervical ulcerated lesion in the present case were attributed to prolonged use of systemic MPZ. Intralesional corticosteroid injections, especially in patients with focal periocular hemangiomas, can be considered an alternative to systemic corticosteroid treatment [2]. Other treatment options include laser, surgical excision, and embolization [6]. We did not treat the presented patient with laser, interferon (IF), or chemotherapy because of the cost and side effects. 

Interferon a-2a and α-2b have become therapeutic options due to their anti-angiogenic and antineoplastic effects, especially in steroid-resistant cases. Ezekowitz et al. [9] reported that 90% of their cases with life-threatening hemangiomas exhibited marked regression after receiving IF a-2a subcutaneously; however, neurologic disturbances, such as spastic diplegia and motor development impairment, limit the widespread use of IF [9]. Furthermore, fever, malaise, neutropenia, anemia, increase in liver transaminases, anorexia, weight loss, confusion, and insomnia have been reported to occur following IF administration [9]. Another limitation of IF is its high cost, as compared to other therapeutic options, especially in countries with limited financial resources. Antineoplastic agents such as vincristine and cyclophosphamide were reported to be effective in treating life-threatening hemangiomas that are corticosteroid-resistant, especially lesions associated with Kasabach-Merritt phenomenon [10,11]. 

The non-selective β blocker propranolol was recently reported to be effective in treating children with cutaneous or laryngotracheal hemangiomas [3,4,5,6,7,8]. Propranolol is primarily used to treat hypertension, and in pediatric cardiology it is used to treat tachycardia and arrhythmias [2]. Vasoconstriction, decreased expression of vascular endothelial growth factor and β-fibroblast growth factor genes, and triggering apoptosis in capillary endothelial cells are among propranolol’s proposed mechanisms of action in regressing hemangiomas [12,13]. Additionally, β2 receptor expression in the endothelial cells of hemangiomatous lesions was reported [13]. Due to the side effects of propranolol, including bradycardia, hypotension, bronchoconstriction, hyperkalemia, and reduced physiological response to hypoglycemia, it should be used cautiously in infants, and routine cardiologic assessment with ECG, ECHO, and monitorization of serum electrolytes and blood glucose levels is recommended before and during the use of propranolol [14,15]. On the other hand, the optimal duration of propranolol treatment remains problematic due to the risk of recurrence when it is withdrawn before the age of spontaneous regression (generally accepted as ≤18 months) [16]. In the presented case, propranolol was withdrawn when the patient was age 13 months and subsequently recurrence developed. 

In the presented patient, we observed acute regression of hemangiomatous lesions and improvement in ulceration following the addition of propranolol to the treatment regimen. The drug was tolerated well and no side effects were observed during the treatment. In some cases, propranolol results in rapid and effective shrinkage of lesions, particularly in the subglottic region [6]. Michel and Patural [17] reported that they successfully treated an ulcerated cutaneous hemangioma with propranolol. In conclusion, propranolol may be considered a treatment option in neonatal patients with ulcerated hemangiomas, particularly in problematic regions such as the head and neck, and in those with subglottic hemangiomas. 

**Conflict of Interest Statement **

The authors of this paper have no conflicts of interest, including specific financial interests, relationships, and/ or affiliations relevant to the subject matter or materials included.
